# Effectiveness and Safety of Acupuncture for Perimenopausal Depression: A Systematic Review and Meta-Analysis of Randomized Controlled Trials

**DOI:** 10.1155/2020/5865697

**Published:** 2020-01-19

**Authors:** Xiao Xiao, Jiayuan Zhang, Yuxia Jin, Yunxia Wang, Qi Zhang

**Affiliations:** Chengdu University of Traditional Chinese Medicine, No. 37 Shierqiao Road, Jinniu District, Chengdu, Sichuan 610075, China

## Abstract

**Objective:**

To determine the effectiveness and safety of acupuncture for perimenopausal depression.

**Methods:**

We searched the Cochrane Central Register of Controlled Trials, PubMed, EMBASE, CNKI, VIP Citation Databases, Wan Fang, and online trial registries such as ClinicalTrials.gov for randomized controlled trials (RCTs) assessing the efficacy and safety of acupuncture for perimenopausal depression. Literature screening, data extraction, and determination of the risk of bias were performed by two researchers independently. The extracted data were pooled and meta-analyzed using RevMan5.3 software.

**Results:**

In total, 16 RCTs covering 1311 patients were enrolled. Overall, the results showed that acupuncture was more effective in the treatment of perimenopausal depression than antidepressants (OR = 2.68, 95% CI (1.84, 3.90), *P* < 0.00001). Furthermore, HAMD scores in the manual acupuncture group and electroacupuncture group were lower than those of antidepressants (manual acupuncture vs. antidepressants (MD = −2.35, 95% CI (−2.93, −1.77), *P* < 0.00001) and electroacupuncture vs. antidepressants (MD = −1.2, 95% CI (−1.92, −0.48), *P*=0.001)). Data analysis revealed that the treatment effect of acupuncture was more stable than that of antidepressants (MD = −2.4, 95% CI (−3.37, −1.43), *P* < 0.00001). Moreover, acupuncture was safer than antidepressants based on the incidence of adverse events (OR = 0.23, 95% CI (0.1, 0.52), *P*=0.0004). But acupuncture has no effect on estrogen levels (*P* ≥ 0.05).

**Conclusions:**

Acupuncture for perimenopausal depression is safe and effective. Moreover, it has more stable long-term effects than antidepressants and hormone replacement therapy (HRT). We recommend acupuncture as a clinical treatment of perimenopausal depression.

## 1. Introduction

Perimenopausal women are at a higher risk of developing depression and other undesirable changes than at any other stage of women's lives [1, 2]. The prevalence of perimenopausal depression is estimated to range between 4.7% and 41.8% [3–5]. Perimenopausal women are known to undergo numerous physiological changes that negatively impact their health physically and mentally. The onset of perimenopause is characterized by feelings of depression, despair, and sometimes suicidal thoughts. It is frequently accompanied by dysfunction of the endocrine and autonomic nervous systems, leading to hot flashes, night sweats, sleeping difficulty, reduced sex drive, and fluctuations in weight, energy, and cognitive ability [6].

However, the underlying causes of high-incidence perimenopausal depression are still unclear [7]. An increasing body of evidence suggests that the elevated risk of depression in perimenopausal women is at least in part, due to hormonal fluctuations, although this theory remains controversial [8–11]. Predisposing factors include sociodemographic factors, psychosocial factors, onset of menopausal symptoms, as well as marital and family status [6]. Conventionally, perimenopausal depression is managed pharmacologically using antidepressants or through psychotherapy [6]. Unfortunately, these therapeutic options are not always effective. Antidepressants are frequently associated with numerous negative side effects including mouth dryness, fatigue, drowsiness, weight gain, and sexual dysfunction, thereby contributing to low patient compliance rates [12, 13]. Hormone replacement therapy (HRT) has also been used in the management of perimenopausal depression, but the effectiveness of this strategy remains uncertain [14, 15]. Therefore, safer and more effective therapeutic options are therefore needed for the management of this condition.

As a promising and effective therapy, acupuncture is gaining widespread global acceptance as a therapeutic option for the management of various health conditions [16]. It has been reported that depression ranks second in the top 10 indications for acupuncture [17]. Moreover, multiple studies and clinical trials have documented the effectiveness of acupuncture in the treatment of depression [18–21]. It should, however, be noted that the physiological-based perimenopausal depression is characterized by wide hormonal fluctuations, therefore differing from general depression [6]. Recent reports from randomized controlled trials (RCTs) on acupuncture indicate that the use of acupuncture in the management of for perimenopausal depression has also risen gradually risen [22].

In this study, we carried out a systematic review of published RCTs on the use of acupuncture in the management of perimenopausal depression, aiming to better understand its benefits in the management of this condition relative to conventional treatment options.

## 2. Methods

### 2.1. Protocol and Registration

This systematic review was registered in PROSPERO as CRD42018114506 and was carried out following a previously described protocol [22]. In brief, the systematic review and meta-analysis was done in accordance with the guidelines outlined in the Cochrane Handbook for Systematic Reviews of Intervention [23] and reported in accordance with PRISMA guidelines [24]. Because this was a review of published studies and did not directly involve patients, ethical clearance and patient consents were not sought.

### 2.2. Study Selection (Inclusion and Exclusion Criteria)

#### 2.2.1. Types of Studies

In this study, we included all RCTs on the use of acupuncture in the management of perimenopausal depression, whether the trials were blinded or not.

#### 2.2.2. Types of Participants

Studies on perimenopausal women diagnosed with depression were included. In this study, the perimenopausal phase was defined using the 2012 criteria described by the North American Menopause Society [25]. Diagnosis of depression in the studies analyzed met at least one of the following criteria: ICD-10 [26], DSM-5™ [27], and CCMD-3 [28]. Studies that included patients with other serious diseases, such as heart disease, stroke, and severe gynecological diseases, were excluded from our analyses.

#### 2.2.3. Types of Interventions

Only studies in which the only intervention within the experimental group was acupuncture (manual or electroacupuncture) were included in our analyses. Studies in which the experimental group was subjected to ear acupuncture, moxibustion, or pharmacologic interventions were not included in our analyses. Studies were included in which the control group had been subjected to various interventions including pharmacologic agents, placebo, and sham acupuncture and treatment courses of greater than 2 weeks. Other conditions, including nursing care, were consistent between the experiment and control groups.

#### 2.2.4. Types of Outcome Measures

Our analysis evaluated primary and secondary outcomes of acupuncture. The primary outcome computed two measures: the effective rate and the Hamilton depression scale (HAMD) score. As previously described, a HAMD score reduction rate ≥25% was regarded as an effective treatment [29]. The lower the HAMD score, the higher effect the interventions had.

The secondary outcome was measured by evaluating the levels of estrogen (FSH, E2, and LH), the HAMD score during follow-up period, and the incidence of adverse events.

### 2.3. Literature Search

Literature searches were done in the following databases: the Cochrane Central Register of Controlled Trials (CENTRAL), PubMed, EMBASE, China National Knowledge Internet (CNKI), Chongqing VIP (CQVIP), Wan Fang Data, as well as in online clinical trial registries such as ClinicalTrials.gov, European Medicines Agency, and the WHO's International Clinical Trials Registry Platform. The searches were conducted to include all relevant records for up to October 2018. No language restrictions were imposed when conducting the literature search. The literature search terms are as illustrated in Table 1.

### 2.4. Study Selection and Data Extraction

Results from the literature search were evaluated by two reviewers (XX and JY Z), who independently determined which articles met the inclusion as described in Section 2.2. Any disagreements and conflicting opinions about which studies to include were resolved by consensus with a third independent reviewer (YX J). Next, XX and JY Z extracted and entered the data from the selected studies into a predefined data template. The extracted data included the study features, information about participants, intervention to which participants were subjected, outcome measures, adverse outcomes, and participants follow-up.

### 2.5. Assessment of Risk of Bias and Quality of Evidences

To minimize reviewer bias, two researchers, XX and YX W, carried out independent evaluations of all the selected studies using a previously described collaboration tool recommended by the Cochrane Handbook [23]. There were six points that had to be evaluated: random allocation, allocation concealment, blinding, incomplete outcome data, selective outcome reporting, and other biases. The disagreement was also resolved by discussion. Finally, funnel plots were used to evaluate publication bias in the more than 10 selected studies as described before [30].

### 2.6. Data Analyses

#### 2.6.1. Date Synthesis

Meta-analysis and data synthesis were carried out using RevMan5.3.5 software from Cochrane collaboration (http://www.cochrane.org). For analyses of dichotomous variables, we used risk ratios (OR) and 95% confidence intervals (95% CI). For analyses of continuous variables, we used mean differences (MD) and 95% confidence. For continuous variables with different units, standardized mean differences (SMD) and 95% confidence intervals (95% CI) were used. *P* values of *P* < 0.05 were considered to be statistically significant.

#### 2.6.2. Assessment of Heterogeneity

The Chi-squared test and the *I*^2^ statistic were used for the determination of heterogeneity. In cases of low heterogeneity, within the acceptable range of *P* > 0.10, *I*^2^ < 50%, the fixed effect model was used for data analysis. Otherwise, the random effect model was used.

#### 2.6.3. Subgroup Analysis and Sensitivity Analysis

In order to evaluate the possible sources of heterogeneity, subgroup analysis and sensitivity analyses were conducted. Subgroup analyses looked at the possible factors that might contribute to heterogeneity, such as intervention in the experimental group, control group, the duration of treatment or the standards of the reports under consideration.

## 3. Results

### 3.1. Study Selection

**Figure 1:**
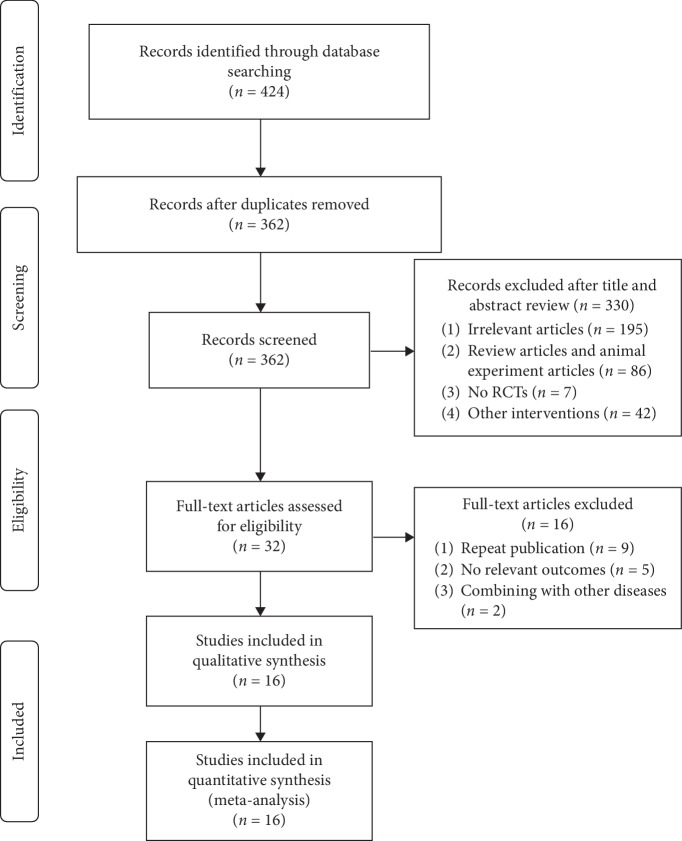
A flowchart of the study selection process.

Following the literature search, 424 studies were identified. Of these, 119 were published in English and 305 in Chinese. Following elimination of duplicates, 362 articles remained for further analyses. Further analyses were conducted by rigorously reviewing the articles' abstracts and bodies to ensure that they met the inclusion criteria and were of sufficient quality. This process eliminated 346 articles leaving 16 [31–46] that were subjected to downstream analyses. Of these, 3 were dissertations [34, 36, 45] and 13 were journal articles (Figure 1).

### 3.2. Study Characteristics

All 16 RCTs that met the criteria for further analyses were conducted in China [31–46]. Of these, 14 reports are published in Chinese [33–46] and 2 in English [31, 32]. One of the studies reported findings of a multicenter RCT [32], while the others reported findings from monocenter RCTs. Together, a total of 1,311 patients were part of our analyses. A total of 660 patients from the experimental groups had undergone acupuncture treatment. Among them, 211 patients from 4 trials [32, 42, 43, 45] had undergone electroacupuncture treatment, while the remaining patients received manual acupuncture treatment. In the control group of the studies we analyzed, all 651 patients had been treated with pharmacological agents. Of the RCTs included in our analyses, control groups in 2 trials [34, 44] were treated with Deanxit, control groups in 8 trials [31, 33, 36–41] with fluoxetine, control groups in 3 trials [35, 45, 46] with fluoxetine in combination with HRT, and the control groups in 3 trials [32, 42, 43] with escitalopram. The duration of treatment ranged between 2 weeks and 12 weeks.

Of the RCTs we analyzed, 14 reported the effective rate [31, 33–39, 41–46] and 14 reported HAMD [32–36, 38–46]. Baseline and difference values of HAMD scores were reported in one of the included trials [32], so we calculated the final HAMD score using the equation recommended by the Cochrane Handbook [23] as follows. Estrogen levels were reported in 2 trials [32, 45] and adverse events in 9 trials [32–34, 36, 39, 40, 44–46] (Table 2).

(1) $$\begin{array}{c} {\text{SD}}_{\text{change}}-\sqrt{{\text{SD}}_{\text{baseline}}^{2}+{\text{SD}}_{\text{final}}^{2}-\left(2r\times {\text{SD}}_{\text{baseline}}\times {\text{SD}}_{\text{final}}\right)}=0. \end{array}$$

### 3.3. Risk of Bias in Included Studies

**Figure 2:**
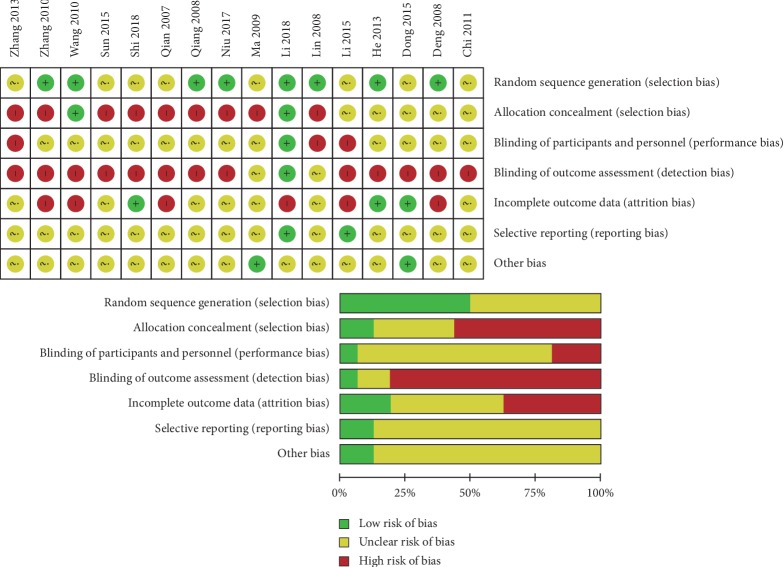
Risk of bias assessment (“+” = low risk, “−” = high risk, and “?” = unclear).

Of the 16 RCTs we analyzed, 8 did not report their methods of generating random sequences, and only 2 trials reported that allocation process was hidden. Because the analyzed RCTs focused on acupuncture, it was not possible to blind the acupuncturists. Only one of the RCTs studies was blinded to patients and to data evaluators. For the other studies, we did bias risk assessments based on implementation methods reported in the respective articles. Only 6 trials reported the reasons for participant withdrawal and dropout. Together, these factors made it hard to establish the integrity or validity of the reported outcomes. The risk of selective reporting and other bias was not found (Figure 2).

### 3.4. Effectiveness of Acupuncture

#### 3.4.1. Effective Rate

**Figure 3:**
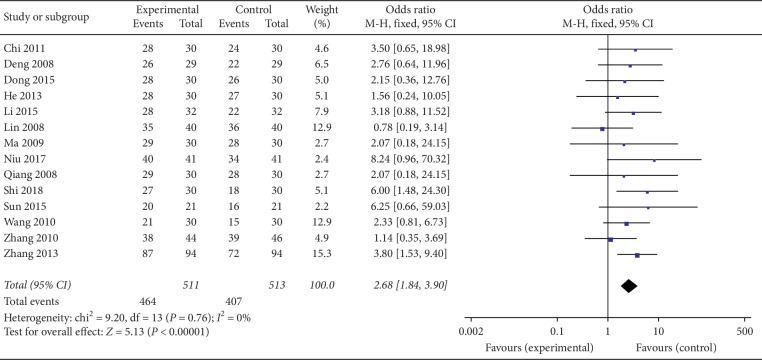
Forest plot for effective rate.

Of the 16 RCTs that we analyzed, 14 reported the effective rates [31, 33–36, 38–46]. Of these 14, the experimental groups in 3 trials [42, 43, 45] had undergone electroacupuncture, while those in the remaining 11 trials [31, 33–39, 41, 42, 46] underwent manual acupuncture. An analysis of the heterogeneity between the studies revealed a remarkably low level of heterogeneity (*I*^2^ = 0%, *P*=0.76). We therefore used the fixed effect model for combination. Our meta-analysis indicated that participants with perimenopausal depression benefited from both acupuncture and drugs. Moreover, our results suggest that acupuncture was more effective than pharmacological agents at alleviating the symptoms of perimenopausal depression (OR = 2.68, 95% CI (1.84, 3.90), *P* < 0.00001) (Figure 3).

#### 3.4.2. HAMD Scores

**Figure 4:**
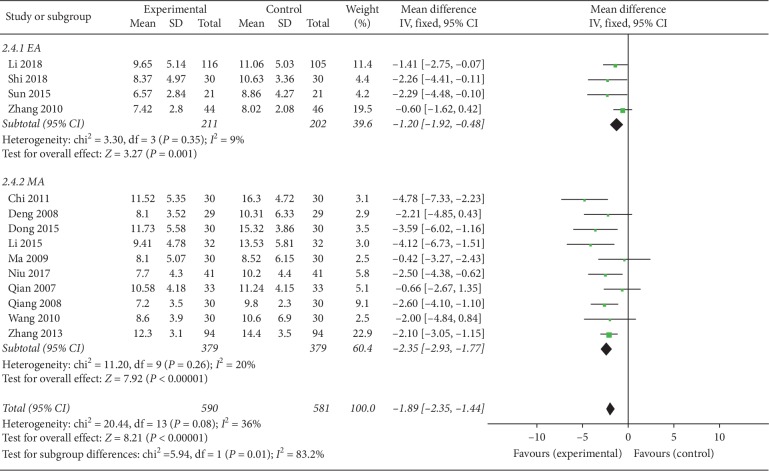
Forest plot for HAMD scores of subgroups (EA: electroacupuncture; MA: manual acupuncture).

Out of the 16 RCTs we analyzed, 14 reported HAMD scores [32–36, 38–46]. Participants in the experimental groups in 4 studies had undergone electroacupuncture, and the remainder manual acupuncture. Results of our meta-analysis revealed significantly lower HAMD scores for the acupuncture groups (*P* < 0.05) (MD = −1.89, 95% CI (−2.35, −1.44), *P* < 0.00001). However, the heterogeneity analysis revealed a slight degree of heterogeneity (*I*^2^ = 36%, *P*=0.08), making it difficult to establish the reliability. To evaluate the factors underlying the low heterogeneity, we conducted subgroup analysis on the different intervention measures (electroacupuncture and manual acupuncture). This analysis revealed the heterogeneity of both manual acupuncture and electroacupuncture groups decreased (Figure 4).

*(1) Electroacupuncture*. Electroacupuncture was used on the experimental groups of 4 trials [32, 42, 43, 45]. Our meta-analysis revealed significantly lower HAMD scores for the electroacupuncture (experimental) group relative to the control group (*P* < 0.05) (MD = −1.2, 95% CI (−1.92, −0.48), *P*=0.001). Because the heterogeneity analysis revealed low heterogeneity (*I*^2^ = 9%, *P*=0.35), the fixed effect model was adopted.

*(2) Manual acupuncture*. Out of the 16 RCTs we analyzed, the experimental groups in 10 underwent manual acupuncture [33–36, 38–41, 44, 46]. Results from our meta-analysis revealed significantly lower HAMD scores in the manual acupuncture (experimental) groups relative to the control groups (*P* < 0.05) (MD = −2.35, 95% CI (−2.93, −1.77), *P* < 0.00001). Because the heterogeneity analysis revealed a low level of heterogeneity (*I*^2^ = 20%, *P*=0.26), the fixed effect model was adopted.

#### 3.4.3. Estrogenic Hormone

**Figure 5:**
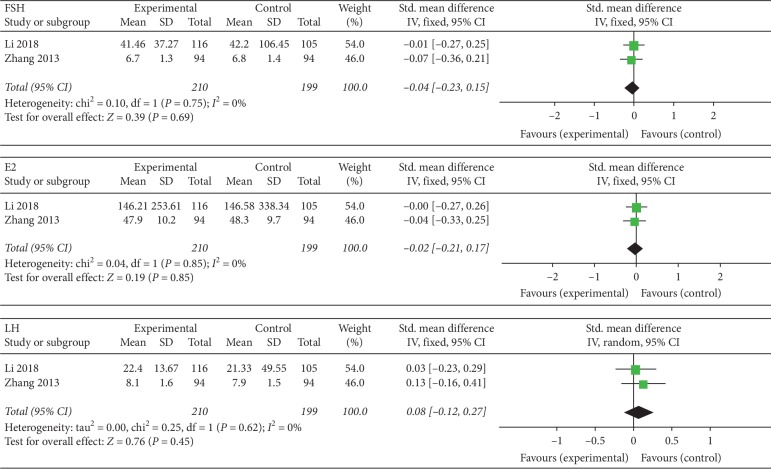
Forest plot for the estrogen levels.

Two RCTs reported having evaluated estrogen levels [32, 45]. The experimental groups in the 2 studies had undergone electroacupuncture. Because of the strikingly low levels of heterogeneity for FSH (*I*^2^ = 0%, *P*=0.75), E2 (*I*^2^ = 0%, *P*=0.85), and LH (*I*^2^ = 0%, *P*=0.62) between the two studies, the fixed effect model was used. But there was no statistical significance of all indexes (*P* ≥ 0.05): FSH (SMD = 0.04, 95% CI (0.23, 0.15), *P*=0.69), E2 (SMD = 0.02, 95% CI (0.21, 0.17), *P* > 0.85), and LH (SMD = 0.08, 95% CI (0.12, 0.41), *P* > 0.45). According to Section 2.6, SMD was adopted due to different data units (Figure 5).

#### 3.4.4. HAMD Scores of the Follow-Up Period

**Figure 6:**
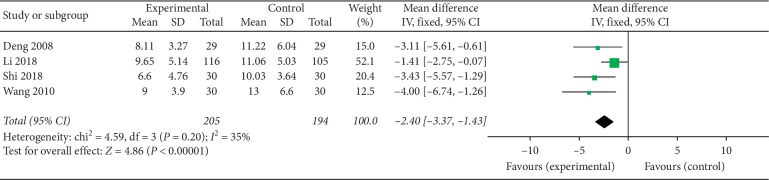
Forest plot for HAMD scores in follow-up period.

Among the 16 RCTs we analyzed, 5 reported follow-up analysis [32, 34, 36, 42, 44], while 4 reported HAMD scores [32, 34, 42, 44]. Because follow-up was carried out for different durations, we based our analysis of follow-up on the data for the fourth week of follow-up in the respective studies. This analysis revealed that the HAMD scores of the acupuncture (experimental) groups were significantly lower than in the control groups (*P* < 0.05) (MD = −2.4, 95% CI (−3.37, −1.43), *P* < 0.00001). Because meta-analysis revealed mild heterogeneity between the 4 trials (*I*^2^ = 35%, *P*=0.02) (an acceptable level of heterogeneity), the fixed effect model was not adopted (Figure 6).

### 3.5. Safety of Acupuncture

**Figure 7:**
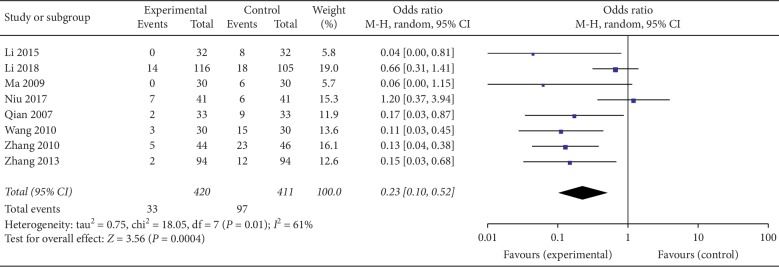
Forest plot for safety of acupuncture.

Of the 16 RCTs that we analyzed, 1 reported the treatment emergent symptom scale (TESS) score [38], while 9 others reported adverse events [32–34, 36, 39, 40, 44–46]. As shown in Table 2, none of the studies reported serious adverse outcomes in the acupuncture (experimental) group or the control. However, it was difficult to estimate adverse outcomes in 2 trials [33, 34], and hence they excluded from further analysis. This analysis revealed that the incidence rate of adverse outcomes in patients treated with acupuncture was significantly lower than in the group treated with pharmacological agents (*P* < 0.05) (OR = 0.23, 95% CI (0.1, 0.52), *P*=0.0004). Because meta-analysis revealed moderate heterogeneity (*I*^2^ = 61%, *P*=0.01) between the studies, the randomized control model was adopted (Figure 7).

### 3.6. Publication Bias

**Figure 8:**
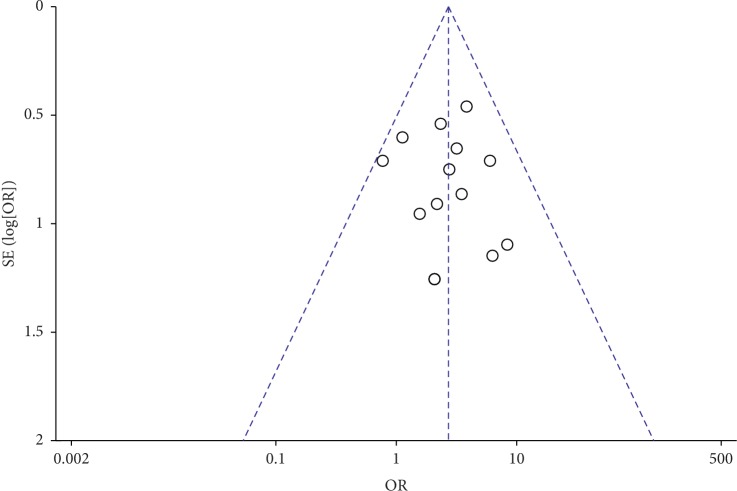
Funnel plot of effective rate.

**Figure 9:**
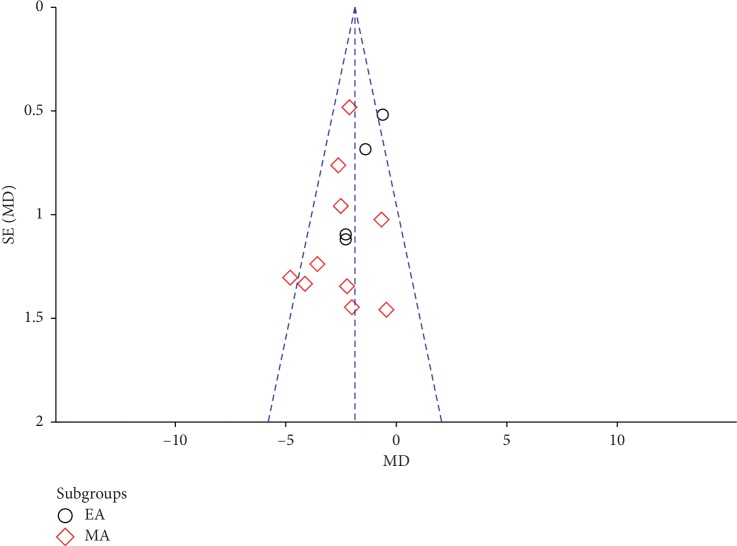
Funnel plot of HAMD scores (EA: electroacupuncture; MA: manual acupuncture).

Because our review and meta-analysis looked at more at more than 10 RCTs, we conducted a publication bias analysis by visual analysis of funnel plots. This analysis produced an almost symmetrical funnel plot distribution, indicating that there was no publication bias (Figures 8 and 9).

## 4. Discussion

### 4.1. Summary of the Main Results

Here, we report a meta-analysis of 16 RCTs that together, comprising 1311 participants. Our analysis of both the effective rate and HAMD score revealed that acupuncture achieved better outcomes in the management of perimenopausal depression-relative antidepressants and HRT. Additionally, follow-up analysis indicates that the benefits of acupuncture against perimenopausal depression last longer those of antidepressants. However, measurements of the levels of estrogenic hormones did not reveal statistically significant differences in those treated by acupuncture relative to pharmacologic antidepressants. Further research is therefore warranted to establish whether the observed acupunctural benefits are associated with changes in the levels of estrogen [47]. In traditional Chinese medicine (TCM), it is theorized that abnormal flow of Qi in a meridian is related to disease and that its natural flow is restorable through acupuncture at appropriate acupoints [48]. Multiple studies suggest that acupuncture has a modulatory, normalizing effect on the limbic-paralimbic-neocortical network (LPNN) of the brain [49–52]. It is generally believed that the LPNN is related to sleep and emotions. Other studies also report that the antidepressant effect of acupuncture is significant and has a multitarget characteristic [53]. Acupuncture treats nervous system diseases by increasing the brain-derived neurotrophic factor level and involving multiple signal pathways, which may be related to amino acid metabolism and inflammatory pathways, especially the toll-like receptor signaling pathway [54].

Our meta-analysis suggests that, in the management of perimenopausal depression, acupuncture is relatively safer than pharmacological agents. The main adverse effects following acupuncture include subcutaneous bleeding and pain following the piercing. These side effects can be reduced by choosing different acupoints. Additionally, patients with fear of needles experience needle sickness, which is characterized by anxiety, dizziness, palpitations, and sweating. These side effects are temporary and can be reduced by training acupuncturists to psychologically prepare patients prior to starting the procedure. On the contrary, negative side effects of antidepressants are related to the respective drugs' mechanisms of action and most cannot be avoidable. It should, however, be noted that the risks associated with acupuncture could not be assessed with confidence as many of the RCTs in our study did not adequately report adverse effects of their interventions.

Our analysis of heterogeneity between the RCT studies we reviewed, revealed mild heterogeneity (HAMD scores of therapeutic effect: *I*^2^ = 36%, *P*=0.08). Subsequent subgroup analysis, in which interventions were separated into manual acupuncture and electroacupuncture, exposed a markedly lower level of heterogeneity between the studies. Mild heterogeneity was also observed following assessment of the effectiveness of treatment during the follow-up period (*I*^2^ = 35%, *P*=0.02). The underlying causes of heterogeneity might be caused by differences in interventions. In addition, the HAMD score is a subjective metric, and results are easily influenced by artificial errors. A safety evaluation revealed moderate heterogeneity (*I*^2^ = 61%, *P*=0.01), and the underlying causes might reflect the assessment method used and/or acupuncturist's professional skill.

### 4.2. Characteristics and Limitations

We are aware of three previous systematic reviews (two in Chinese and one in English) addressing this topic [55–57]. However, these studies did not carry out meta-analysis, and the inclusion criteria were not sufficiently rigorous, causing high levels of heterogeneity between studies. This has made it difficult to accurately evaluate the safety and effectiveness of acupuncture. Unlike previous systematic reviews, we evaluated more recent clinical trials. In addition, we applied stricter inclusion and exclusion criteria, used widely accepted analyses tools, and performed meta-analysis. In order to limit our analysis to the use of body acupuncture in the management of perimenopausal depression, we limited the intervention in the experimental group to manual acupuncture and electroacupuncture only and excluded the studies on ear acupuncture, moxibustion, or pharmacologic interventions. We also evaluated the durability of acupunctural benefits over the follow-up period as well as the effects of the length treatment period. Moreover, we evaluated the safety of perimenopausal interventions by computing the incidence rate of adverse effects.

The RCTs included in our analyses had several shortcomings including the generation of random numbers, distribution hiding, and blind methods. Of the studies we evaluated, only one was registered in ClinicalTrials.gov [32]. It was therefore difficult to estimate the risk of bias in this study. All the included studies were conducted in China. Moreover, we did not carry out manual retrieve, and the collection of gray literature might be insufficient. Participants in the control groups of the RCTs we analyzed had been treated with antidepressants. None of the studies that we evaluated reported the use of placebo or sham acupuncture, limiting our ability to more confidently evaluate the effectiveness of acupuncture. However, this is a defect in the design of the RCTs we included, and it is also a place that needs to be improved in future clinical trials.

### 4.3. Conclusion

Our analysis indicates that the use of acupuncture in the management of perimenopausal depression is safe, effective, and offers long-lasting benefits relative to antidepressants and HRT. Our data indicate that acupuncture is an effective option for the clinical management of perimenopausal depression. Because the existing studies are not of sufficiently high quality, higher quality RCTs are required to establish the clinical benefits and long-term effectiveness of acupuncture in the treatment of perimenopausal depression.

## Figures and Tables

**Table 1:** Search strategy used in PubMed database.

No.	Search terms
**#1**	“Perimenopause”[Mesh]
**#2**	((((((menopaus*∗*[tiab]) OR Perimenopausal[tiab]) OR premenopaus*∗*[tiab]) OR postmenopaus*∗*[tiab]) OR climacteri*∗*[tiab]) OR involution*∗*[tiab]) OR midlife[tiab]
**#3**	#1 OR #2
**#4**	“Depression”[Mesh]
**#5**	((depressi*∗*[tiab]) OR melancholia*∗*[tiab]) OR blue[tiab]
**#6**	#4 OR #5
**#7**	#3 AND #6
**#8**	“Acupuncture”[Mesh]
**#9**	((((electro?acupuncture[tiab]) OR needl*∗*[tiab]) OR acupoint*∗*[tiab]) OR acupuncture point*∗*[tiab]) OR *∗*acupuncture[tiab]
**#10**	#8 OR #9
**#11**	(clinical[tiab] AND trial[tiab]) OR “clinical trials as topic”[mesh] OR “clinical trial”[pt] OR random*∗*[tiab] OR “random allocation”[mesh] OR “therapeutic use”[sh]
**#12**	#7 AND #10 AND #11

**Table 2:** Characteristics of included RCTs.

First author, year	Sample size		Period of treatment	Experimental intervention		Control intervention	Outcome measures	Adverse events (*n*)		Follow-up times
	Active group	Control group		Type	Main acupuncture points			Active group	Control group	
Chi 2011	30	30	4 weeks	MA	DU20, EX-HN3, EX-HN1, LR-14, LR3, KI3, ST36, SP6	Prozac	Effective rate, HAMD reduction rate, HAMD score, 5-HT	Occasional subcutaneous hematoma	Dizziness (1), nausea (2)	NR
Deng 2008	29	29	4 weeks	MA (AA)	CV12, CV10, RN6, RN4, RN3, KI17 (left)	Deanxit	Effective rate, HAMD reduction rate, HAMD score, KMI score	Changes of character of stool (2), palpitation (1)	Dry mouth and halitosis (9), dysphoria (6), changes of character of stool (6), dreaminess (6), breast distending pain (5)	The 6th week and the 8th week
Dong 2005	30	30	1 month	MA	BL13, BL14, BL17, BL18, BL13, BL21, BL23	HRT+ Prozac	Effective rate, HAMD reduction rate, HAMD score	NR	NR	NR
He 2013	30	30	8 weeks	MA (AA)	CV12, CV10, RN4, RN6, KI13, Qipang (0.5 cun beside RN 6)	Prozac	Effective rate, HAMD reduction rate, BDI score	NR	NR	NR
Li 2015	32	32	3 months	MA	BL23, BL18, BL14, DU20, EX-HN1, DU24, EX-HN3, PC6	Prozac	Effective rate, HAMD reduction rate, HAMD score, KMI score	None	Nausea, vomiting, dry mouth, indigestion, diarrhea, insomnia, headache, dizziness (8)	After 3 months
Lin 2008	40	40	6 weeks	MA	DU20, EX-HN3, PC6, HT7, LI4, LR3, SP6, KI3	Prozac	Effective rate, HAMD reduction rate	NR	NR	NR
Li 2018	116	105	12 weeks	EA	RN4, EX-CA1, ST25, SP6, LI4, LR3, DU20, EX-HN3	Escitalopram	HAMD score, MENQOL score, FSH, E2, LH	Subcutaneous hematoma (14),	Dizziness, palpitation, stomachache (18)	The 16th week and the 24th week
Ma 2009	30	30	8 weeks	MA	HT7, PC7, DU20, EX-HN3, PC6, EX-HN1, ST36, SP6	Prozac	Effective rate, HAMD reduction rate, HAMD score, TESS score	None	Dizziness (2), nausea (4)	NR
Niu 2017	41	41	6 weeks	MA	BL13, BL14, BL17, BL18, BL13, BL23	Prozac	Effective rate, HAMD reduction rate, HAMD score	Dizziness (2), palpitation (1), dry mouth (1), nausea (3)	Dizziness (1), palpitation (2), dry mouth (2), nausea (1)	NR
Qiang 2008	30	30	4 weeks	MA	EX-HN1, GB20, BL14, BL18, BL23	Prozac	Effective rate, HAMD reduction rate, HAMD score	NR	NR	NR
Qian 2009	33	33	6 weeks	MA	BL13, BL14, BL17, BL18, BL13, BL23	Prozac	HAMD score	Dizziness (2), palpitation (1)	Insomnia (1), akathisia (1), dry mouth (1), nausea (1), palpitation (1), skin symptom (1), excitement (2)	NR
Shi 2018	30	30	12 weeks	EA	RN4, EX-CAI, ST25, SP6, LI4, LR3, DU20, EX-HN3	Escitalopram	Effective rate, HAMD reduction rate, HAMD score	NR	NR	The 16th week and the 24th week
Skn 2015	21	21	12 weeks	EA	RN4, EX-CAI, ST25, SP6, LI4, LR3, DU20, EX-HN3	Escitalopram	Effective rate, HAMD reduction rate, HAMD score	NR	NR	NR
Wang 2010	30	30	4 weeks	MA (AA)	CV12, CV10, RN6, RN4, RN3, KI17 (left)	Deanxit	Effective rate, HAMD reduction rate, HAMD score	Palpitation (1) changes of character of stool (2)	Dry mouth and bitter mouth (9), breast distending pain, dreaminess, dysphoria (6)	The 6th week and the 8th week
Zhang 2010	44	46	3 months	EA	DU20, PC6, LR3, KI3, SP6, BL13, BL14, BL18, BL13, BL23	HRT+ Prozac	Effective rate, HAMD reduction rate, HAMD score, KMI score, FSH, E2, LH	Sweating, dizziness, vomiting (5)	Dry mouth and halitosis (5), nausea (6), dysphoria (2), constipation (6), dreaminess (2), breast distending pain (2)	NR
Zhang 2013	94	94	3 months	MA	SP6, BG13, DU24, EX-HN1, HT7	HRT+ Prozac	Effective rate, HAMD reduction rate, HAMD score, FSH, E2, LH	Feeling pain when inserting needle (2)	Dizziness (5), nausea and vomiting (4), hypersomnia (3)	NR

EA, electroacupuncture; MA, manual acupuncture; AA, abdominal acupuncture; HRT, hormone replacement therapy; HAMD, Hamilton depression scale; MENQOL, menopause-specific quality of life questionnaire; BDI, back depression inventory; KMI, Kupperman index; NR, not reported.

## Abbreviations

HAMD: Hamilton depression scale
OR: Risk ratio
MD: Mean difference
SMD: Standardized mean difference
CI: Confidence interval.
